# Reversal of diabetes-associated cognitive impairment through modulation of BDNF, insulin, and NF-κB pathways by a marketed herbal formulation

**DOI:** 10.1007/s13205-026-04827-7

**Published:** 2026-05-28

**Authors:** Nabeel Kinattingal, Venugopal Bovilla, Seema Mehdi, Mahadevaswamy G. Kuruburu, K. Nandakumar, Santhepete N. Manjula

**Affiliations:** 1 Department of Pharmacology, JSS College of Pharmacy, JSS Academy of Higher Education and Research, Mysuru, Karnataka 570015 India; 2 Department of Biological Science, North Carolina State University, Raleigh, NC USA; 3 Center of Excellence in Molecular Biology and Regenerative Medicine Laboratory, Department of Biochemistry, JSS Medical College, JSS Academy of Higher Education and Research, Mysuru, 570015 Karnataka India; 4 Department of Pharmacology, Manipal College of Pharmaceutical Sciences, Manipal Academy of Higher Education, Udupi, Karnataka 576104 India

**Keywords:** Blood glucose, Cognitive decline, Diabetes mellitus, Hippocampus, Marketed herbal formulation, *Tamarindus indica* L. seed extract

## Abstract

Diabetes-associated cognitive impairment is a recognized complication of chronic hyperglycemia affecting learning and memory. We investigated whether *Tamarindus indica* seed extract (TSE) and Diabecon, a marketed herbal formulation (MHF), could reverse diabetes-associated cognitive decline in a high-fat diet (HFD) and streptozotocin (STZ) induced type 2 diabetic rat model. Diabetic rats were treated with pioglitazone (standard), TSE (250 mg/kg), or MHF (500 mg/kg) for four weeks. Antioxidant activity and phenolic content of TSE and MHF were evaluated using DPPH radical scavenging and ferric-reducing antioxidant power (FRAP) assays, and animals were assessed for learning and memory using the Barnes maze and novel object recognition tests. Hippocampal biochemical analysis revealed reduced antioxidant levels, decreased BDNF and insulin levels, and increased oxidative stress and inflammatory cytokines in diabetic rats. Behavioral assessments demonstrated significant cognitive impairment in HFD–STZ diabetic rats, evidenced by increased escape latency time and reduced preference index. Treatment with MHF significantly improved cognitive performance compared with untreated diabetic animals, whereas TSE showed limited effects. Biochemical analysis revealed that diabetes induction reduced hippocampal BDNF and insulin levels and increased oxidative stress and inflammatory cytokines. MHF treatment significantly restored BDNF and insulin levels, reduced lipid peroxidation, enhanced endogenous antioxidant enzyme activity, and suppressed inflammatory mediators. Western blot analysis further demonstrated modulation of NF-κB and Nrf2 signaling pathways in the hippocampus following MHF treatment. These findings suggest that the marketed herbal formulation Diabecon may alleviate diabetes-associated cognitive decline by reducing oxidative stress and inflammation while improving neurotrophic signaling.

## Introduction

Diabetes mellitus (DM) is a global health concern, and complications associated with uncontrolled hyperglycemia warrant continued research to develop improved therapeutic and preventive strategies.

In addition to risks of cataracts, retinopathy, neuropathy, and other microvascular complications, DM affects the hippocampus, which has a significant role in learning and memory, thus affecting cognitive functions (Biessels and Despa 2020). Several studies have demonstrated a direct association between diabetes mellitus and cognitive impairment, which in severe or progressive cases may contribute to the development of dementia (Southorn and Powis 1988; Kinattingal et al. 2023; Sheen and Sheu 2016; Davì et al. 2005). Cognitive impairment is increasingly recognized as a complication of diabetes alongside retinopathy, neuropathy, nephropathy, and cardiovascular risk (Kodl and Seaquist 2008).

Although the mechanisms underlying cognitive impairment in diabetes are not fully understood, oxidative stress is known to play a key role in its onset and progression (Rösen et al. 2001). For instance, cognitive impairment in diabetic rat models is associated with increased reactive oxygen species (ROS) levels and reduced antioxidant defences (Zilliox et al. 2016). In other reports, the administration of antioxidants has been shown to reverse cognitive impairment in diabetic rats (Wang and Jia 2014; Tian et al. 2016). Chronic hyperglycemia and microvascular disease contribute to cognitive impairment in diabetes mellitus, including deficits in learning, memory, and attention, and are associated with mental and psychomotor slowing as well as reduced executive function (Cox et al. 2005).

Increasing evidence indicates that oxidative stress, neuroinflammation, and impaired neurotrophic signaling play critical roles in diabetes-associated cognitive decline. Reduced levels of brain-derived neurotrophic factor (BDNF) have been linked with hippocampal neuronal dysfunction and impaired synaptic plasticity in diabetic conditions. Similarly, disruption of insulin signaling within the brain has been associated with impairments in neuronal energy metabolism and synaptic function, leading to cognitive impairment. In addition, activation of inflammatory pathways such as nuclear factor kappa B (NF-κB) contributes to neuroinflammation and neuronal damage in diabetes-related neurodegeneration. Therefore, agents capable of modulating oxidative stress, inflammatory signaling, and neurotrophic pathways may provide therapeutic benefits in preventing or reversing diabetes-associated cognitive impairment. Furthermore, emerging evidence suggests that certain antidiabetic therapies may also help prevent or mitigate diabetes-associated cognitive impairment (Alagiakrishnan et al. 2013; Zhong et al. 2018).

Based on accumulating evidence linking type 2 diabetes mellitus (T2DM) with cognitive impairment, we induced T2DM in rats to evaluate the effects of uncontrolled hyperglycemia on cognitive decline. The animals were then treated with an antioxidant-rich extract of *Tamarindus indica* seed (TSE) and Diabecon, a marketed herbal formulation (MHF), to assess the reversibility of cognitive impairment.

*Tamarindus indica* seed extract (TSE) is known for its antidiabetic potential, and its anti-obesity properties have been demonstrated in a recent study (Nabeel et al. 2020). Diabecon is a polyherbal formulation widely used in the management of type 2 diabetes mellitus and contains several plant-derived components with reported antioxidant, anti-inflammatory, and antidiabetic activities (Grover et al. 2002; Patel et al. 2012). Previous studies have demonstrated that such phytochemicals can influence molecular pathways involved in neuroprotection, including regulation of oxidative stress responses and inflammatory mediators (Vauzour et al. 2008; Spencer et al. 2012). However, despite its widespread use as an antidiabetic formulation, its potential effects on diabetes-associated cognitive decline and underlying neurobiological mechanisms remain largely unexplored.

Based on the established association between oxidative stress, inflammation, and neurotrophic signaling in diabetes-related cognitive impairment, we hypothesized that antioxidant-rich interventions may attenuate hippocampal neurodegeneration and improve cognitive performance in diabetic conditions. We investigated the effects of *Tamarindus indica* seed extract (TSE) and the marketed herbal formulation Diabecon (MHF) on diabetes-associated cognitive decline in a high-fat diet and streptozotocin-induced type 2 diabetic rat model.

## Materials and methods

### Reagents and chemicals

Folin–Ciocalteu reagent (FCR) (Cat. No.: F9252, Merck, USA), gallic acid (Cat. No.: G7384, Merck, USA), and quercetin (Cat. No.: Q4951, Merck, USA) were purchased from M/s. Merck, USA. 2,2-diphenyl-1-picryl-hydrazyl (DPPH) (Cat. No.: D9132, Sigma-Aldrich, USA), HRP-conjugated secondary antibody (Cat. No.: 12–349; RRID: AB_390192; Sigma-Aldrich, USA), phosphate-buffered saline (PBS) (Cat. No.: P3813, Sigma-Aldrich, USA), and Tris-buffered saline (TBS) (Cat. No.: T6664, Sigma-Aldrich, USA) were used in the study. RIPA buffer (Cat. No.: 89900, Thermo Fisher Scientific, USA), Pierce BCA Protein Assay Kit (Cat. No.: 23227, Thermo Fisher Scientific, USA), and β-actin antibody (clone AC-15; Cat. No.: AM4302; RRID: AB_2536382; Thermo Fisher Scientific, USA) were also obtained.

The ELISA kits for TNF-α (Cat. No.: SEA133Ra), IL-6 (Cat. No.: SEA079Ra), and IL-1β (Cat. No.: SEA563Ra) were procured from Cloud-Clone Corp., USA. The primary antibodies anti-Nrf2 (Cat. No.: PAL947Ra01), anti-NQO1 (Cat. No.: PAL969Ra01), anti-NF-κB (Cat. No.: PAB824Ra01), and anti-BDNF (Cat. No.: PAA011Ra01)—were obtained from Cloud-Clone Corp., USA (RRIDs not available). The Insulin ELISA kit (Cat. No.: A05105, Bertin Pharma, France) was used as obtained.

Diabecon^®^ tablets (marketed herbal formulation, MHF) were purchased from Himalaya Wellness Company, Bengaluru, India. According to the manufacturer’s label, the formulation contains multiple herbal ingredients traditionally used for glycemic control, including Gymnema sylvestre, Momordica charantia, Pterocarpus marsupium, Tinospora cordifolia, Syzygium cumini, and Emblica officinalis, among others. The tablets were finely powdered and suspended in 0.5% carboxymethyl cellulose (CMC) prior to oral administration to animals. The formulation used in the study was obtained from a single manufacturing batch to minimize potential batch-to-batch variability during the experimental study.

### Animals

Male Sprague Dawley (SD) rats weighing 180–200 g were used in this study. Animals were obtained from a CPCSEA registered breeder—In vivo Biosciences, Bengaluru, Karnataka, India. The Institutional Animal Ethics Committee (IAEC) approval was obtained before the commencement of the study (Approval number: 198/2016&251/2017). All experimental procedures involving animals were conducted in accordance with the ARRIVE (Animal Research: Reporting of In Vivo Experiments) guidelines and relevant national regulations for the care and use of laboratory animals. Throughout the experimental period, animals were regularly monitored for general health status, body weight changes, food intake, and any signs of distress or abnormal behaviour to ensure animal welfare. Animals were acclimatized for 7 days under laboratory conditions (55% humidity; 22–25 °C temperature) and a lighting sequence of 12 h light and 12 h dark cycles. All animals were fed standard rat pellets and water *ad libitum*.

### Tamarindus indica Linn. seed

*Tamarindus indica* Linn. seeds were collected from a single tree in Gundelpet Taluk, Charmaraja District, Mysuru. The sample was verified and authenticated by Dr. Naganandini, Assistant Professor, Department of Pharmacognosy, JSS College of Pharmacy, Mysuru- 570,015, Karnataka (certificate No:289/09/2015/JSSCPM).

### Preparation of Tamarindus indica Linn. seed extract (TSE)

The collected seeds were processed and extracted as per the method of Luzia and Jorge (Sánchez-Rangel et al. 2013). The macerate obtained was pooled and concentrated using a rotary vacuum evaporator (Rotavapor^®^ R-210/215, BÜCHI Labo Technik AG) to obtain a solid mass that was stored at 4 °C until use.

### Quantitative estimation of TSE & MHF

Since Diabecon is a polyherbal formulation containing several plant-derived bioactive compounds such as flavonoids, phenolics, alkaloids, and terpenoids, preliminary phytochemical characterization was performed through estimation of total phenolic content and antioxidant activity assays. These assays provide an indirect indication of the antioxidant potential of the formulation.

#### Total phenolic content

Total phenolic content was estimated in triplicate using the method of Sánchez-Rangel et al. (Sánchez-Rangel et al. 2013). Although detailed phytochemical profiling of the polyherbal formulation was beyond the scope of the present study, estimation of total phenolic content and antioxidant activity was performed to provide an initial indication of its bioactive antioxidant constituents.

### Antioxidant activity of TSE & MHF

#### DPPH scavenging assay

The DPPH assay was performed in triplicate according to the method reported by Waqas et al. (Waqas et al. 2013). The percentage scavenging activity was determined, and the half-maximal inhibitory concentration (IC_50_) was calculated and expressed as µg/ml.

$$\:\mathrm{\%}\:\mathrm{S}\mathrm{c}\mathrm{a}\mathrm{v}\mathrm{e}\mathrm{n}\mathrm{g}\mathrm{i}\mathrm{n}\mathrm{g}\:\mathrm{a}\mathrm{c}\mathrm{t}\mathrm{i}\mathrm{v}\mathrm{i}\mathrm{t}\mathrm{y}=\frac{\mathrm{C}\mathrm{o}\mathrm{n}\mathrm{t}\mathrm{r}\mathrm{o}\mathrm{l}\:\mathrm{a}\mathrm{b}\mathrm{s}\mathrm{o}\mathrm{r}\mathrm{b}\mathrm{a}\mathrm{n}\mathrm{c}\mathrm{e}-\mathrm{S}\mathrm{a}\mathrm{m}\mathrm{p}\mathrm{l}\mathrm{e}\:\mathrm{a}\mathrm{b}\mathrm{s}\mathrm{o}\mathrm{r}\mathrm{b}\mathrm{a}\mathrm{n}\mathrm{c}\mathrm{e}\:}{\mathrm{C}\mathrm{o}\mathrm{n}\mathrm{t}\mathrm{r}\mathrm{o}\mathrm{l}\:\mathrm{a}\mathrm{b}\mathrm{s}\mathrm{o}\mathrm{r}\mathrm{b}\mathrm{a}\mathrm{n}\mathrm{c}\mathrm{e}\:}\times\:100$$

#### FRAP assay

The ferric-reducing antioxidant power (FRAP) assay was performed using a modified method described by Benzie and Strain (Benzie and Strain 1996) to estimate the antioxidant power of TSE and MHF by determining the ability of samples to reduce Fe^3+^ ions into Fe^2+^ ions. Gallic acid was used as a positive control. FRAP units were calculated and expressed as micromolar ferrous sulphate equivalent per milligram of the sample.

### In vivo animal model to study the protective effect of TSE & MHF

#### Induction of diabetes by high-fat diet and streptozotocin

A high-fat diet (HFD) was prepared according to the composition of Vijaimohan et al. (Vijaimohan et al. 2006). Each animal (SD rat), except those in the Normal group, received an HFD of 12 g/day for 4 weeks. Streptozotocin (STZ) was used to induce T2DM using the method reported by Skovsø (Skovsø 2014). The complete study plan is presented in Table 1.

**Table 1:**
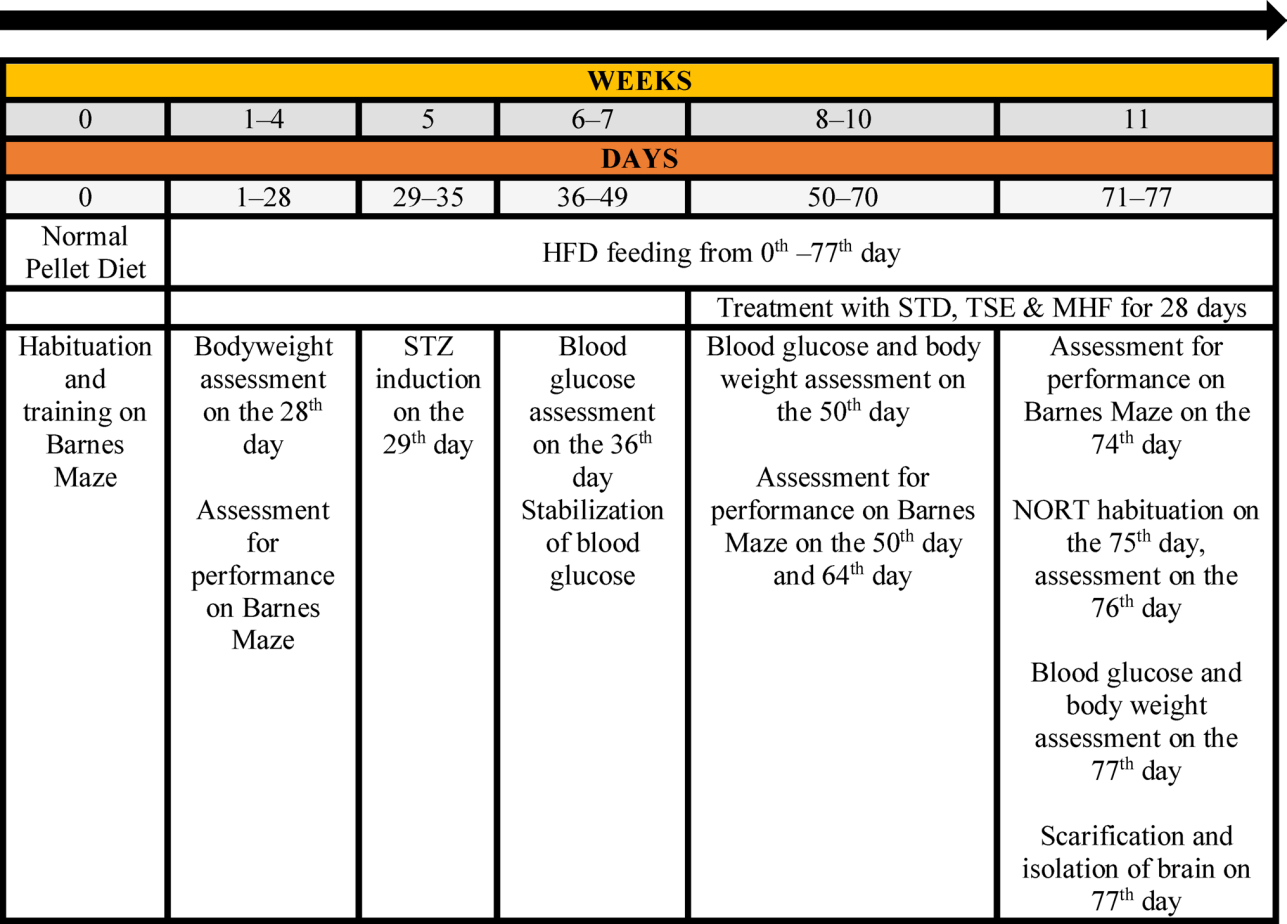
Study Plan for HFD and STZ model

HFD: high-fat diet; MHF: Marketed Herbal Formulation; NORT: novel object recognition test; STD: standard—Pioglitazone; STZ: streptozocin; TSE: *Tamarindus indica* Linn. Seed Extract

On the 50th day of the total 77-day experimental period, animals were randomly allocated into six experimental groups (*n* = 10 per group) to minimize selection bias. The Normal group consisted of age-matched non-diabetic rats that neither received HFD nor STZ.

The doses of Tamarindus indica seed extract (TSE; 250 mg/kg bwt) and the marketed herbal formulation (MHF; 500 mg/kg bwt) were selected based on previously reported pharmacological studies demonstrating their antidiabetic and antioxidant activities in experimental animal models (Nabeel et al. 2020). These doses were chosen to ensure effective pharmacological activity while maintaining safety in rodents.

All other groups received their assigned treatments for 4 weeks (8th–11th week), as presented in Table 2. A treatment duration of four weeks was selected based on previous studies evaluating cognitive and biochemical changes in HFD–STZ diabetic models (Swain et al. 2022; Kaur et al. 2025; Isawi et al. 2026), where similar intervention periods were sufficient to detect improvements in metabolic and neurobehavioral parameters.

**Table 2:** Grouping of animals based on treatment

Group	Treatment 4 Weeks (28 days)
Normal	Normal pellet diet + CMC
HFD control	HFD + CMC
STZ control	HFD + STZ (30 mg/kg bwt, IP)
STD	HFD + STZ (30 mg/kg bwt, IP) + Pioglitazone (20 mg/kg bwt, PO)
TSE	HFD + STZ (30 mg/kg bwt, IP) + TSE (250 mg/kg bwt, PO)
MHF	HFD + STZ (30 mg/kg bwt, IP) + MHF (500 mg/kg bwt, PO)

bwt: bodyweight; CMC: carboxymethyl cellulose; HFD: high-fat diet; IP: intraperitoneal; MHF: Marketed Herbal Formulation; PO: peroral; STD: standard—Pioglitazone; STZ: streptozocin; TSE: *Tamarindus indica* Linn. Seed Extract

#### Bodyweight and blood glucose assessment

Body weight of the animals was monitored throughout the experimental period using a digital weighing scale (Essae-DS-773SS), and blood glucose levels were measured using a glucometer (One Touch II), as described in Table 1. The animals were evaluated for proper induction and stabilization of the glucose levels (200 mg/dl) at the beginning of the 8th week.

#### Assessment of cognitive abilities

The Barnes maze test was performed according to the previously described protocol (Barnes 1979). The time taken by the animal to reach the escape hatch (a target hole connected to a dark escape box) from the start box, known as escape latency time (ELT), was assessed on the 28th, 50th, 64th, and 74th day for each animal. Furthermore, the number of errors was counted by recording each rat’s nose dips to explore the various holes on the surface of the maze. Representative images of the tracking of animals were analyzed by ANYMAZE 6.1 Software on all four days. Behavioral assessments and data analysis were performed by an investigator blinded to the treatment groups to minimize observational bias.

The novel object recognition test (NORT) was performed as reported by Antunes and.

Biala (Antunes and Biala 2012) to measure the preference of animals towards a novel object and a familiar object. The assay was performed 48 h before sacrificing the animals after habituating on the 75th day. The cognitive capabilities of the animal were determined by calculating the preference index (PI), which was calculated as the ratio of the amount of time spent exploring the novel objects in the test phase (B) over the total time spent exploring both familiar objects (A) and novel objects, i.e., B/ (A + B) × 100 (%) in the test phase. Therefore, a PI above 50% indicated novel object preference, a PI below 50% familiar indicated object preference and a PI equal to 50% indicated no preference.

#### Estimation of endogenous antioxidant levels in the hippocampus

The total study duration of 77 days was designed to allow sufficient time for high-fat diet feeding, induction and stabilization of diabetes using streptozotocin, followed by a four-week treatment period to evaluate both metabolic and cognitive outcomes. At the end of the study, on the 77th day, the animals were sacrificed, the hippocampus was isolated, and 10% homogenate was prepared using phosphate buffer (0.2 M) pH 8.0 and subjected to centrifugation. Later, clear supernatant was used for the test of endogenous antioxidant enzyme actions (Pari and Latha 2004).

##### Lipid peroxidation

Lipid peroxidation in the brain was assessed calorimetrically using thiobarbituric acid reactive substances (TBARS) and hydroperoxides by the method of Niehaus and Samuelsson (Niehaus and Samuelsson 1968). The malondialdehyde (MDA) concentration of the sample was calculated using the following formula:

$$\:\frac{\mathrm{A}}{EC}\times\:\mathrm{D}\mathrm{F}=\mathrm{M}\mathrm{D}\mathrm{A}\:{\upmu\:}\mathrm{m}\mathrm{o}\mathrm{l}\times\:1000=\mathrm{M}\mathrm{D}\mathrm{A}\:\mathrm{n}\mathrm{m}\mathrm{o}\mathrm{l}/\mathrm{m}\mathrm{g}\:\mathrm{p}\mathrm{r}\mathrm{o}\mathrm{t}\mathrm{e}\mathrm{i}\mathrm{n}$$

Where, A= Absorbance, EC= Extinction coefficient, DF= Dilution factor.

##### Superoxide dismutase (SOD) activity

The SOD activity was determined according to the method suggested by Kakkar, Das, and Viswanathan (Kakkar et al. 1984). The enzyme activity was expressed in U/mg protein, where 1U is the amount of enzyme required to inhibit the rate of adrenaline auto-oxidation by 50%.

$$\:\frac{\mathrm{C}\mathrm{o}\mathrm{n}\mathrm{t}\mathrm{r}\mathrm{o}\mathrm{l}\:\mathrm{a}\mathrm{b}\mathrm{s}\mathrm{o}\mathrm{r}\mathrm{b}\mathrm{a}\mathrm{n}\mathrm{c}\mathrm{e}-\mathrm{S}\mathrm{a}\mathrm{m}\mathrm{p}\mathrm{l}\mathrm{e}\:\mathrm{a}\mathrm{b}\mathrm{s}\mathrm{o}\mathrm{r}\mathrm{b}\mathrm{a}\mathrm{n}\mathrm{c}\mathrm{e}\:}{\mathrm{C}\mathrm{o}\mathrm{n}\mathrm{t}\mathrm{r}\mathrm{o}\mathrm{l}\:\mathrm{a}\mathrm{b}\mathrm{s}\mathrm{o}\mathrm{r}\mathrm{b}\mathrm{a}\mathrm{n}\mathrm{c}\mathrm{e}}\times\:\mathrm{D}\mathrm{F}=\frac{\mathrm{U}\:\mathrm{p}\mathrm{e}\mathrm{r}\:\mathrm{m}\mathrm{l}\:}{\mathrm{P}\mathrm{r}\mathrm{o}\mathrm{t}\mathrm{e}\mathrm{i}\mathrm{n}}=\mathrm{U}/\mathrm{m}\mathrm{g}\:\mathrm{o}\mathrm{f}\:\mathrm{P}\mathrm{r}\mathrm{o}\mathrm{t}\mathrm{e}\mathrm{i}\mathrm{n}$$

##### Catalase (CAT) activity

CAT activity was determined according to the method of Sinha (Sinha 1972). The enzyme activity was expressed as U/mg protein, where 1U of the enzyme is the amount of enzyme required to inhibit the decomposition rate of H_2_O_2_ by 50% than the assay conditions.

$$\:\frac{2.303}{{\Delta\:}\mathrm{t}}\times\:\mathrm{l}\mathrm{o}\mathrm{g}\frac{\mathrm{A}1}{\mathrm{A}2}\times\:60\:\mathrm{m}\mathrm{i}\mathrm{n}\times\:1000/\mathrm{P}\mathrm{r}\mathrm{o}\mathrm{t}\mathrm{e}\mathrm{i}\mathrm{n}\:=\:\mathrm{U}/\mathrm{m}\mathrm{g}\:\mathrm{o}\mathrm{f}\:\mathrm{P}\mathrm{r}\mathrm{o}\mathrm{t}\mathrm{e}\mathrm{i}\mathrm{n}$$

Where, Δt = 60 min, A1 = absorbance at 0 min, and A2 = absorbance at 60 min.

#### Estimation of neurotransmitters in the hippocampus by HPLC—ECD

Neurotransmitters in hippocampal tissues were estimated by the method of Zandy et al. using the HPLC system (Model 1645, M/s. Waters, USA) with C-18 column (RP- C-18, particle size- 4.6, 5 μm x250 mm at 30 °C) (Zandy et al. 2017). The concentration of neurotransmitters was presented as µg/g of wet tissue.

The isolated hippocampus was homogenized using a Probe Sonicator, set to 30 Pulse, with an amplitude of 70%, and an on-and-off cycle of 2s and 5s, respectively. The homogenized tissue was centrifuged at 12,000 rpm at 4 °C for 10 min, and the supernatant was collected.

Protein estimation was performed for the tissue samples by the Pierce Bicinchoninic acid (BCA) Protein Assay Kit method at 1:8 dilution (protein sample: water). Protein-normalized samples were further used for ELISA and protein expression studies in Western Blot (Wang et al. 2019a, b).

#### Estimation of insulin in hippocampal homogenate by ELISA

Insulin was measured using a commercially available colorimetric ELISA kit (A05105- SPI BIO- Bertin Pharma) according to the manufacturer’s instruction, where acetylcholinesterase (AChE) was used as the enzymatic label for the enzyme immunometric assay (EIA).

#### Estimation of inflammatory cytokines in hippocampal homogenate by ELISA

The pro-inflammatory cytokines TNF-α, IL-6, and IL-1β, were measured using commercially available colorimetric ELISA kits (SEA133Ra, SEA079Ra, and SEA563Ra- Cloud- Clone Corp) according to the manufacturer’s instructions, and their levels were expressed as pg/mg of protein.

#### Protein expression studies by Western blot

The protein expression of NF-кB, Nrf2, NQO1, and BDNF in the hippocampus of rats was detected by Western blot using the protein-normalized samples prepared for ELISA. The study used primary antibodies of Nrf2 (PAL947Ra01- Cloud- Clone Corp), NQO1(PAL969Ra01- Cloud- Clone Corp), NF-κB (PAB824Ra01- Cloud- Clone Corp), and BDNF (PAA011Ra01- Cloud- Clone Corp) (1:800) dilution in TBST buffer, a secondary antibody conjugated with HRP (1:2000) in TBST buffer and (Enhanced Chemiluminescence), and an ECL substrate.

Imaging and data analysis were performed by using chemiluminescence reagents. Chemiluminescence signals were captured using Hitachi Gene Tools CCD BIO software. Densitometric analysis of protein bands was performed, and the relative expression levels of target proteins were normalized to β-actin as an internal loading control.

### Statistical analysis

Data are presented as mean ± standard error of the mean (SEM) of the indicated number of animals. The sample size for behavioral experiments (*n* = 10 per group) was selected based on previous studies using similar HFD–STZ diabetic models assessing cognitive function in rodents (Swain et al. 2022; Xourgia et al. 2019). Prior to statistical comparisons, the distribution of the data was evaluated for normality using the Shapiro–Wilk test. For comparisons among multiple groups, one-way analysis of variance (ANOVA) followed by Tukey’s multiple comparison test was used for biochemical and molecular parameters. For behavioral assessments in the Barnes maze across different time points, two-way ANOVA followed by Bonferroni post hoc test was applied. These post hoc multiple comparison procedures were used to control type I error during multiple group comparisons. Statistical analyses were performed using GraphPad Prism software, and values were considered statistically significant at *p* < 0.05.

## Results

### Total phenolic content

The phenolic content in 1 g of dried sample of MHF was higher (54.37 ± 0.73 µg/ml gallic acid equivalents (GAE)) than in 1 g of dried sample of TSE (45.41 ± 0.75 µg/ml GAE).

### Antioxidant activity of TSE and MHF

#### DPPH scavenging assay

The DPPH assay for estimation of the free radical scavenging ability of TSE & MHF revealed a higher DPPH radical scavenging ability of MHF (74.22 ± 1.6% at 100 µg/ ml) than of TSE (63.75 ± 2.55% at 100 µg/ ml) with an IC_50_ value of 49.83 ± 3.16 µg/ml and 56.47 ± 2.7 µg/ml for MHF and TSE, respectively. The DPPH radical scavenging ability of reference standard ascorbic acid was estimated to be 85.98 ± 0.34% at 100 µg/ ml with an IC_50_ value of 54.82 ± 0.49 µg/ml.

#### FRAP assay of TSE and MHF

The FRAP assay data demonstrated a much higher ferric-reducing ability of MHF (96.91 ± 0.84% inhibition at 100 µg/ml) compared to TSE (81.57 ± 0.21% inhibition at 200 µg/ml).

### Bodyweight

**Fig. 1:**
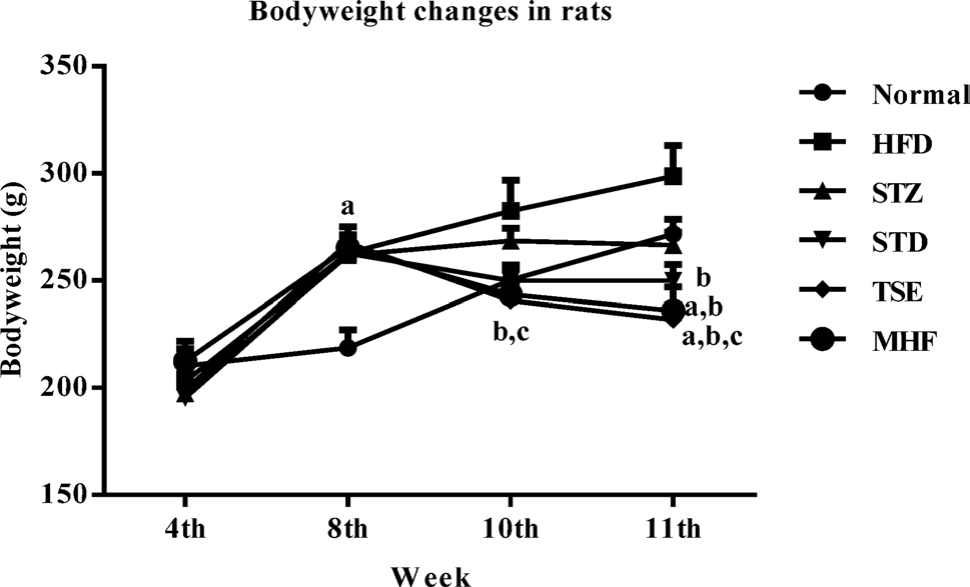
Periodic body weight changes in experimental groups during the study period. Body weight of animals in Normal, HFD control, STZ control, STD (pioglitazone), TSE (Tamarindus indica seed extract), and MHF (Diabecon) groups was recorded at the 4th, 8th, 10th, and 11th weeks of the experimental period. Data are presented as mean ± SEM (*n* = 10 animals per group). Statistical analysis was performed using two-way ANOVA followed by Bonferroni post hoc test. *p* < 0.05: p.a. = vs. Normal group; pb = vs. STZ control group; pc = vs. HFD control group

Changes in body weight of the animals during the experimental period are presented in Fig. 1. On the 50th day, all the HFD-fed animals showed a significant increase in bodyweight compared with the animals in the Normal group. On the 77th day, the animals in MHF (236.0 ± 11.0 g) and TSE (231.8 ± 6.9 g) groups displayed a significant decrease in their body weight compared to animals in Normal (271.6 ± 6.9 g) and STZ (266.4 ± 6.1 g) groups.

*3.4. Evaluation of blood glucose level in diabetes-induced animals before and after treatment with STD*,* TSE & MHF*.

**Fig. 2:**
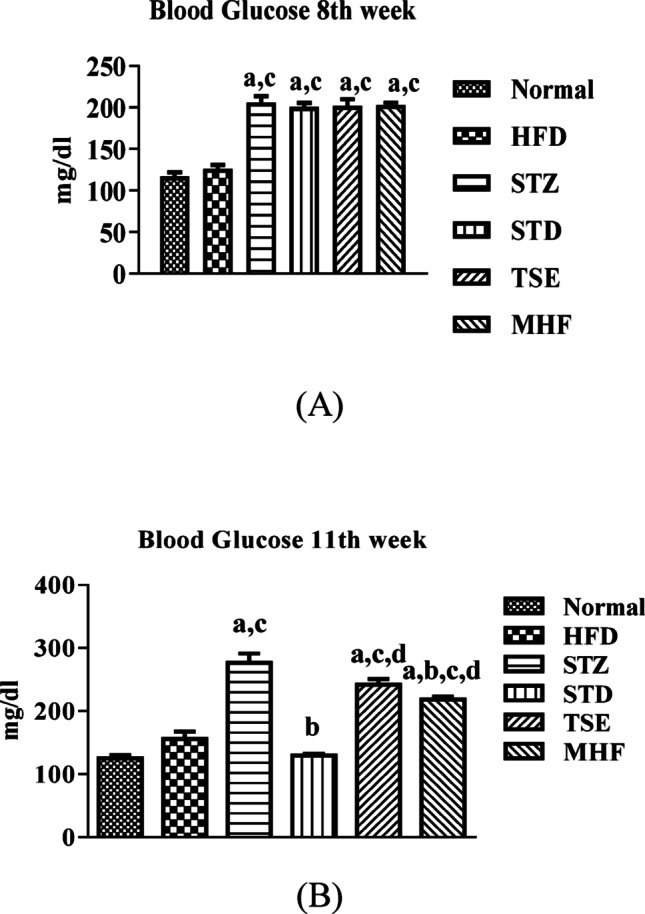
**A** Fasting blood glucose levels following induction of diabetes. Blood glucose levels were measured one week after streptozotocin (STZ) administration (8th week) in Normal, HFD control, STZ control, STD, TSE, and MHF groups to confirm successful induction of hyperglycemia. Values are expressed as mean ± SEM (*n* = 10 animals per group). Statistical analysis was performed using one-way ANOVA followed by Tukey’s post hoc test. *p* < 0.05: *p*a = vs. Normal group; *p*c = vs. HFD group. **B** Fasting blood glucose levels after treatment. Blood glucose levels were measured at the end of the treatment period (11th week) in all experimental groups (Normal, HFD, STZ, STD, TSE, and MHF). Data represent mean ± SEM (*n* = 10 animals per group). Statistical analysis was performed using one-way ANOVA followed by Tukey’s post hoc test. *p* < 0.05: p.a. = vs. Normal group; pb = vs. STZ control group; pc = vs. HFD group; pd = vs. STD group

On the 50th day (8th week), the animals in the HFD control group ( not induced diabetes with STZ), showed only a slight increase in blood glucose compared with the Normal group (HFD: 125 ± 5 mg/dl), but the animals in STZ (205 ± 8 mg/dl), STD (200 ± 5 mg/dl), TSE (202 ± 8 mg/dl), and MHF (203 ± 3 mg/dl) groups showed a significant increase in blood glucose levels compared to the Normal group (117 ± 5 mg/dl). The results are summarized in Fig. 2A.

Blood glucose estimation on the 77th day (11th week) showed a significantly elevated glucose level in the STZ group (277 ± 15 mg/dl) compared to the Normal group. However, as compared to STZ, the STD, TSE, and MHF groups showed a significantly reduced blood glucose level (130 ± 3, 242 ± 9, and 218 ± 5 mg/dl, respectively). Data is summarized and presented in Fig. 2B.

### Evaluation of cognitive abilities in HFD-STZ-induced diabetic animals

**Fig. 3:**
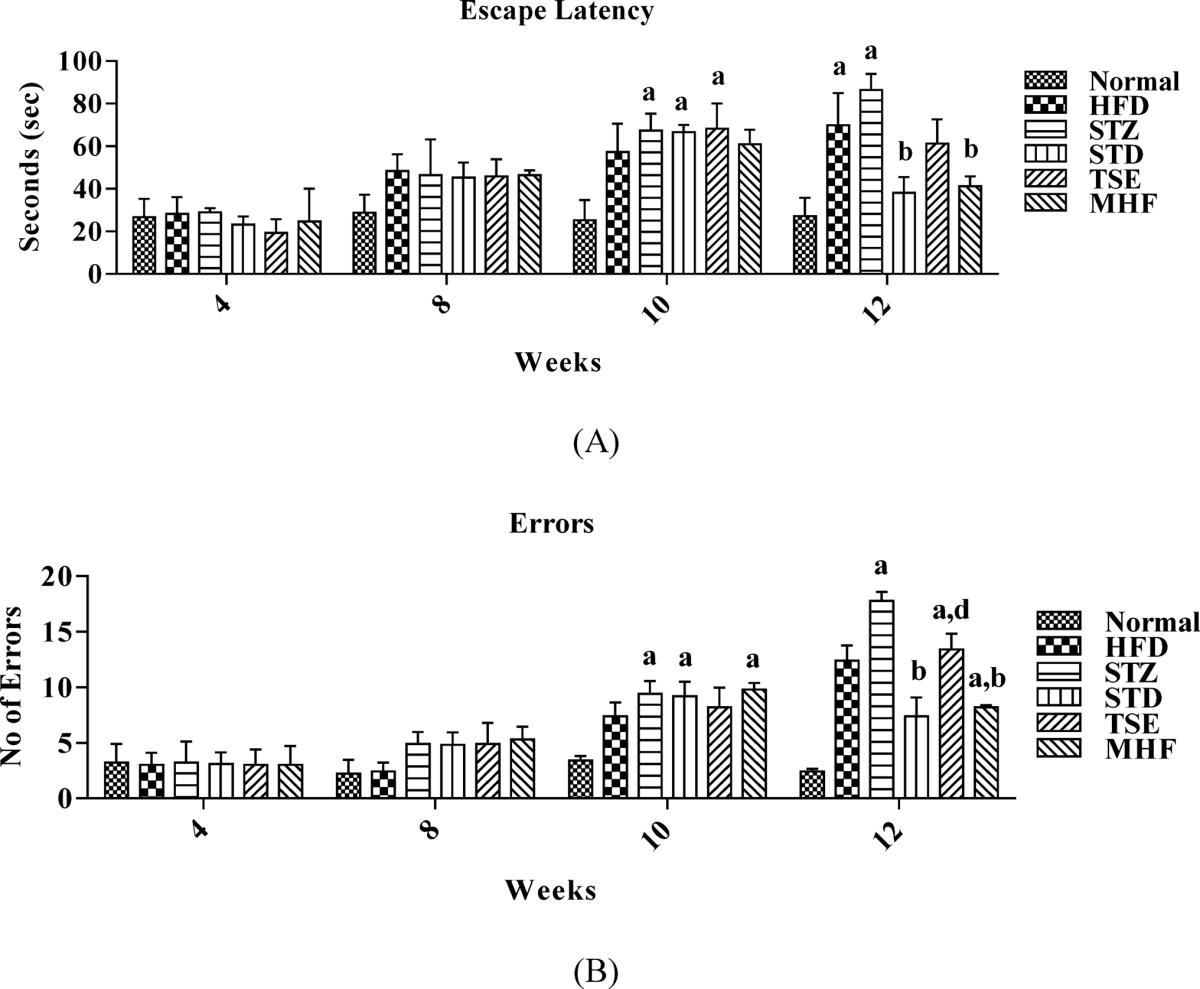
**A** Escape latency time (ELT) in the Barnes maze test. Escape latency time representing spatial learning and memory performance was assessed using the Barnes maze test at the 4th, 8th, 10th, and 11th weeks in Normal, HFD control, STZ control, STD, TSE, and MHF groups. ELT represents the time taken by each animal to locate the escape box. Data are expressed as mean ± SEM (*n* = 10 animals per group). Statistical analysis was performed using two-way ANOVA followed by Bonferroni post hoc test. *p* < 0.05: p.a. = vs. Normal group; pb = vs. STZ control group; pc = vs. HFD group; pd = vs. STD group. **B** Number of errors committed during the Barnes maze test. The number of exploratory errors made by animals before locating the escape hatch was recorded during the Barnes maze test at the 4th, 8th, 10th, and 11th weeks. Increased errors indicate impaired spatial learning. Data are expressed as mean ± SEM (*n* = 10 animals per group). Statistical analysis was performed using two-way ANOVA followed by Bonferroni post hoc test. *p* < 0.05: p.a. = vs. Normal group; pb = vs. STZ control group; pc = vs. HFD group; pd = vs. STD group

Figure 3A and B present the escape latency time (ELT) and the number of errors committed by animals during the Barnes maze test.

The animals in all groups reached the escape hatch within 27 ± 2 s when assessed on the 28th day after training, which increased to 48 ± 8 and 46 ± 17 s for HFD control and STZ groups, respectively, when assessed on the 50th day. Similarly, the animals committed fewer errors (3 ± 2) on the 28th day, which increased moderately (5 ± 1) on the 50th day in all groups except the HFD group.

On the 77th day, STD and MHF treatment groups, but not the TSE treatment group, showed a significant decrease in ELT and the number of errors compared to the STZ and HFD control groups.

### Track map

**Fig. 4:**
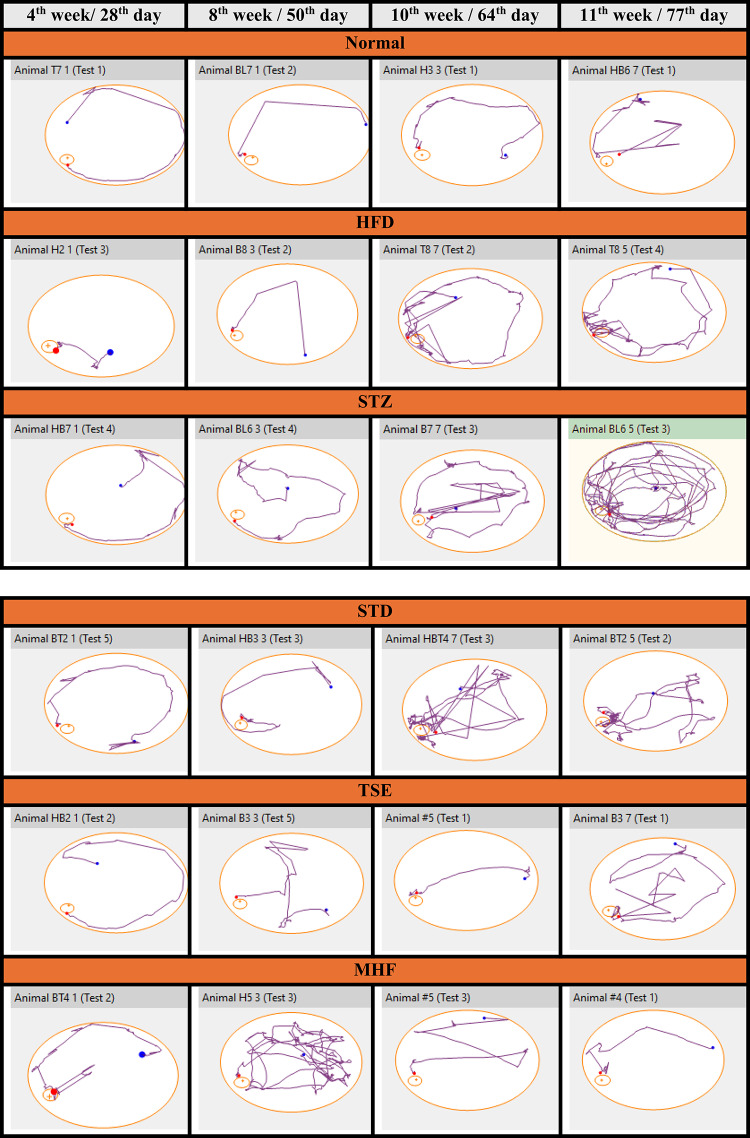
Representative tracking patterns of animals in the Barnes maze test. Representative track maps showing search strategies adopted by animals from Normal, HFD control, STZ control, STD, TSE, and MHF groups during Barnes maze assessment at the 4th (28th day), 8th (50th day), 10th (64th day), and 11th (77th day) weeks. The trajectories illustrate differences in spatial navigation behavior, search strategy complexity, and escape efficiency among experimental groups

Track map images as presented in Fig. 4, revealed that animals from all the groups used simple search strategies to find the escape hatch and committed fewer errors on the 28th day. However, after induction of diabetes by HFD-STZ on the 50th day all the animals except those in the HFD Control group adopted more complicated search strategies.

on the 77th day, the animals treated with STD, TSE, and MHF, demonstrated comparatively simpler search strategies, while the animals in the STZ group showed a progressively complicated search strategy adopted throughout the 8th, 10^th,^ and 11th weeks as it was left untreated.

Interestingly animals in the HFD group, even though not induced with STZ, showed the adoption of a complicated search strategy throughout the study.

### Novel object recognition Test (NORT)

#### Preference index

The animals in the Normal group preferred the novel object 60.42 ± 0.98% of the observed time. Interestingly the animals in the STD group showed a higher preference trend of 66.23 ± 2.74%, and the animals in HFD and STZ showed a substantial decrease in the PI when related to the Normal group. The diabetic animals in the STD, TSE, and MHF showed a substantial increase in the novel object’s preference compared to the STZ group.

### Estimation of endogenous antioxidant levels in the hippocampus

**Fig. 5:**
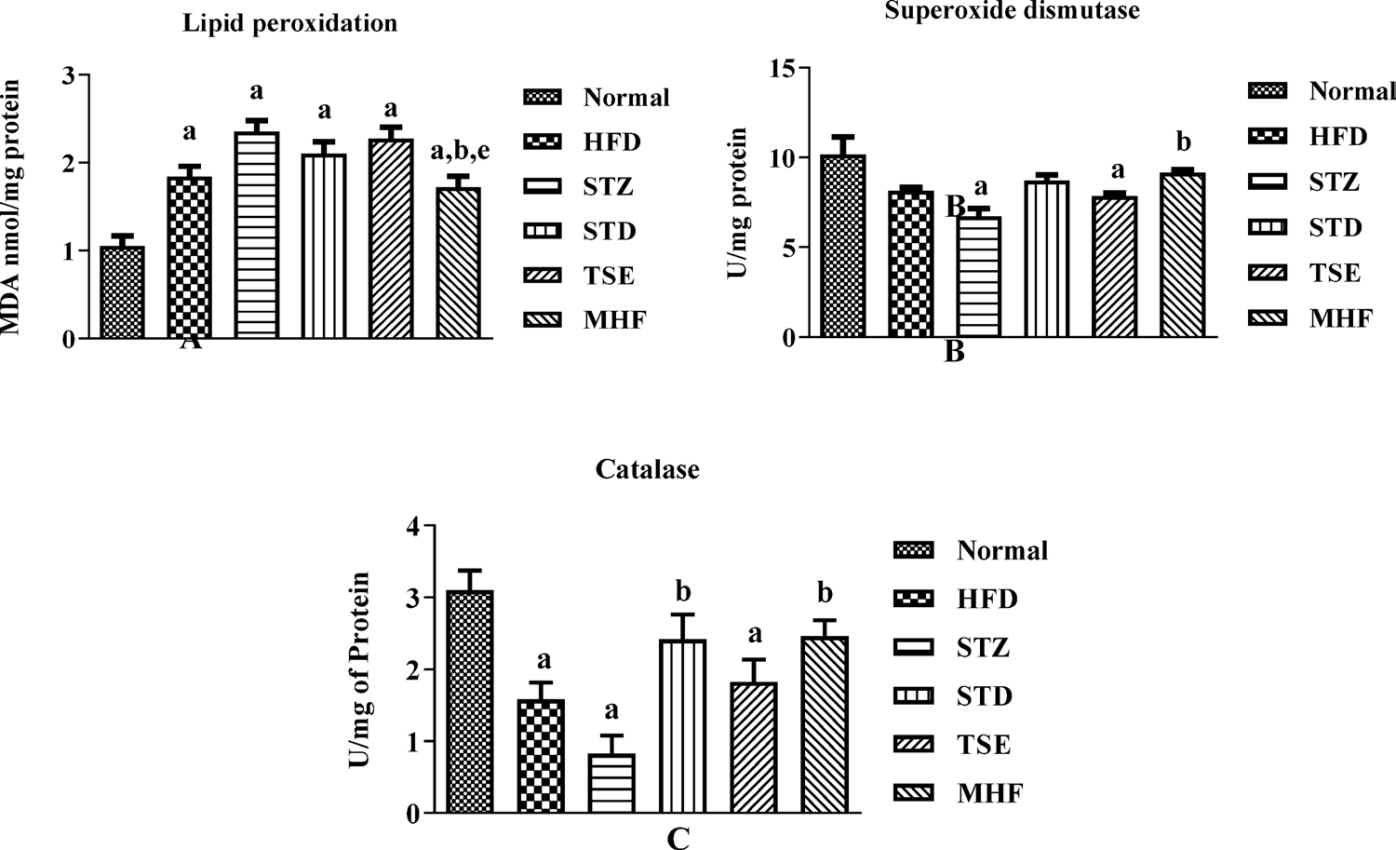
Endogenous antioxidant status in hippocampal tissue. (A) Malondialdehyde (MDA) levels indicating lipid peroxidation, (B) superoxide dismutase (SOD) activity, and (C) catalase (CAT) activity was measured in hippocampal homogenates of Normal, HFD control, STZ control, STD, TSE, and MHF groups. Data are expressed as mean ± SEM (*n* = 3 biological replicates per group). Statistical analysis was performed using one-way ANOVA followed by Tukey’s post hoc test. *p* < 0.05: *p*a = vs. Normal group; *p*b = vs. STZ group; *p*e = vs. TSE group

The results of MDA levels and SOD and CAT activity, as estimated in the hippocampus of rats are presented in Fig. 5A and C. All the animals presented a marked increase in MDA (1.72 ± 0.12–2.36 ± 0.12 nmol/mg protein) when compared to the Normal group (1.05 ± 0.11 nmol/mg protein), while there was a substantial decrease in the MHF group (1.72 ± 0.12 nmol/mg protein) when equated to the STZ (2.36 ± 0.12 nmol/mg protein) group. A decreasing trend nonsignificant to the STZ group was observed in the animals in the STD (2.10 ± 0.13 nmol/mg protein) and TSE group (2.27 ± 0.13 nmol/mg protein). There was a significant elevation in the lipid peroxidation in the HFD group (1.84 ± 0.12 nmol/mg protein) compared to the Normal group.

The animals in STZ and TSE showed a significant decrease in SOD levels (6.70 ± 0.45 and 7.84 ± 0.16 U/mg protein respectively) when compared with the Normal group (10.16 ± 0.9731 U/mg protein); while there was a substantial increase in SOD levels in the MHF group (9.14 ± 0.18 U/mg protein) as compared to the STZ group. An increasing trend was observed for the animals in the STD group (8.71 ± 0.31 U/mg protein), but it was not significant to the STZ group.

All animals except those in STD and MHF group showed a significant decrease in CAT levels when compared with the Normal group (3.10 ± 0.27 U/mg protein). At the same time, there was a substantial increase in the MHF (2.46 ± 0.22 U/mg protein) and STD group.

(2.41 ± 0.35 U/mg protein) when compared with the STZ group (0.83 ± 0.25 U/mg protein). An increasing trend was observed for the animals in the TSE group (1.82 ± 0.31 U/mg protein), but it was not significant to the STZ group. Interestingly, there was a substantial decrease in the catalase levels of the HFD group (1.58 ± 0.24 U/mg protein) compared to the Normal group.

### Estimation of neurotransmitter levels in the hippocampus of animals

**Fig. 6:**
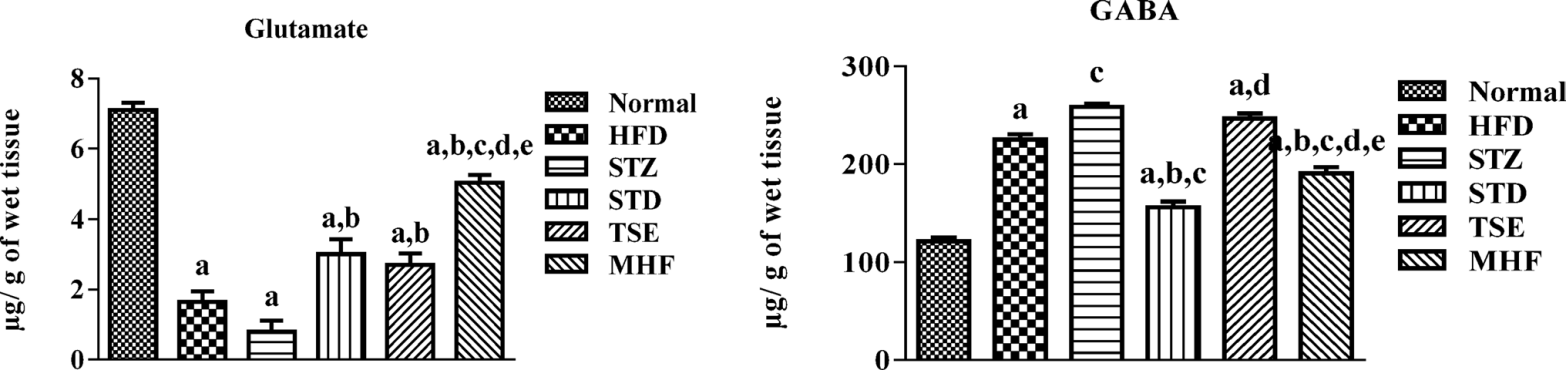
Neurotransmitter levels in hippocampal tissue. (A) Glutamate and (B) γ-aminobutyric acid (GABA) concentrations were quantified in hippocampal homogenates to assess alterations in excitatory and inhibitory neurotransmission in the HFD–STZ diabetic model. Data are expressed as mean ± SEM (*n* = 3 biological replicates per group). Statistical analysis was performed using one-way ANOVA followed by Tukey’s post hoc test. *p* < 0.05: *p*a = vs. Normal group; *p*b = vs. STZ group; *p*e = vs. TSE group

The processed data of neurotransmitters level are presented in Fig. 6A and B.

A significant decrease in glutamate levels was observed across groups compared to the Normal group (7.10 ± 0.21 µg/g of wet tissue), while a noteworthy increase was observed in the STD (3.01 ± 0.42 µg/g of wet tissue), TSE (2.70 ± 0.32 µg/g of wet tissue), and MHF.

(5.03 ± 0.22 µg/g of wet tissue) groups compared to the STZ group (3.01 ± 0.42 µg/g of wet tissue).

A noteworthy increase in GABA levels was observed compared to the Normal group (121.46 ± 4.10 µg/g of wet tissue). An increasing trend was also observed in the STZ group (258.45 ± 3.22 µg/g of wet tissue) although it was not significant. A substantial decrease was observed in the STD and MHF groups (156.12 ± 6.16 and 190.42 ± 6.44 µg/g of wet tissue, respectively) compared to the STZ group.

### Estimation of insulin levels in the hippocampus of animals using ELISA

The animals in the STZ group showed a noteworthy decrease in insulin levels when related to the Normal group (22.42 ± 3.29 in STZ vs. 47.20 ± 4.83 ng/mg in Normal). A decreasing trend was observed in the HFD (34.60 ± 3.34 ng/mg) and TSE group (34.70 ± 4.46 ng/mg), but it was not significant to the Normal group. There was a substantial increase in the STD and MHF groups (45.83 ± 1.63 and 58.96 ± 8.24 ng/mg respectively) compared to the STZ group.

### Estimation of inflammatory cytokines levels in the hippocampus of animals

**Fig. 7:**
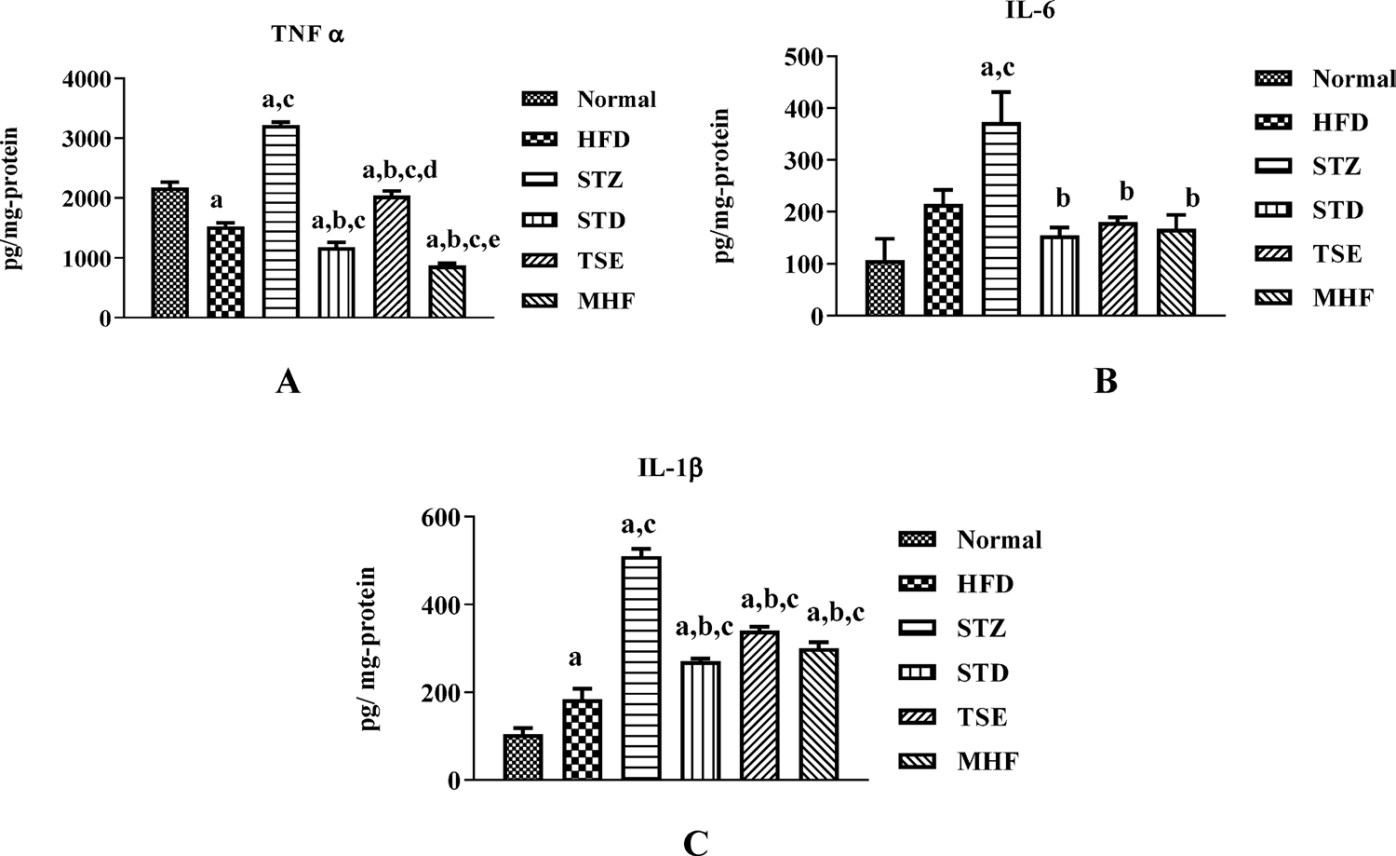
Pro-inflammatory cytokine levels in hippocampal tissue. Levels of (A) TNF-α, (B) IL-6, and (C) IL-1β were quantified in hippocampal homogenates to evaluate neuroinflammatory responses in HFD–STZ-induced diabetic rats and the effect of treatments. Data are expressed as mean ± SEM (*n* = 2 biological replicates per group). Statistical analysis was performed using one-way ANOVA followed by Tukey’s post hoc test. *p* < 0.05: p.a. = vs. Normal group; pb = vs. STZ group; pc = vs. HFD group; pd = vs. STD group; pe = vs. TSE group

The data for the estimation of inflammatory markers is captured in Fig. 7A and C.

A substantial decrease in TNF-α levels was observed compared to the Normal group (2176.67 ± 89.60 pg/mg of protein), whereas a significant increase was observed in the STZ group (3215.56 ± 53.55 pg/mg of protein). Furthermore, the STD, TSE, and MHF groups showed a marked decrease in TNF-α levels (1176.67 ± 82.50, 2037.78 ± 80.67, and 871.11 ± 39.28 pg/mg of protein, respectively) compared to the STZ group.

Animals in the STZ group showed a significant increase in IL-6 levels (373.33 ± 57.31 pg/mg of protein) when compared with the Normal (106.67 ± 41.25 pg/mg of protein), as well as those in STD, TSE, and MHF groups (153.89 ± 15.71, 179.78 ± 9.82, 166.67 ± 27.50 pg/mg protein respectively).

A substantial increase in IL-1β levels was observed compared to the Normal group (103.5 ± 14.14 pg/mg of protein), with a marked increase in the STZ group (510.0 ± 16.94 pg/mg of protein). The STD, TSE, and MHF groups showed a significant decrease in IL-1β levels (270.0 ± 7.071, 340.0 ± 8.84, and 300.0 ± 14.14 pg/mg of protein, respectively) compared to the STZ group.

###  Protein expression studies by Western blot in the hippocampus of diabetic animals

**Fig. 8:**
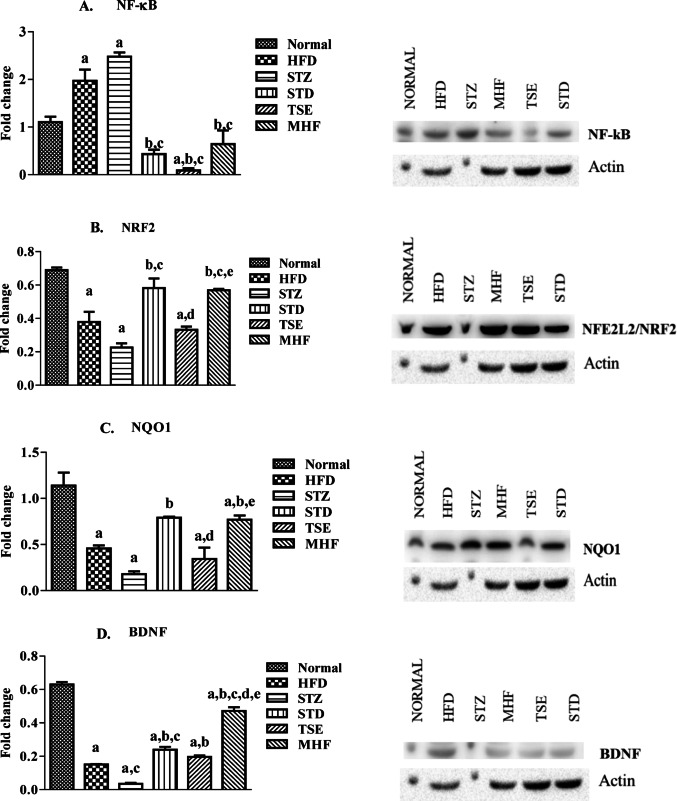
Western blot analysis of signaling pathway proteins in hippocampal tissue. Relative protein expression levels of (A) NF-κB, (B) Nrf2, (C) NQO1, and (D) BDNF were analyzed in hippocampal tissue from Normal, HFD control, STZ control, STD, TSE, and MHF groups using Western blotting. Representative blot images are shown along with densitometric quantification normalized to β-actin. Data are presented as mean ± SEM (*n* = 2 biological replicates per group). Statistical analysis was performed using one-way ANOVA followed by Tukey’s post hoc test. *p* < 0.05: p.a. = vs. Normal group; pb = vs. STZ group; pc = vs. HFD group; pd = vs. STD group; pe = vs. TSE group

Data analyses of protein expression studied by western blot in the hippocampus are presented in Fig. 8A and C.

The animals in the HFD and STZ groups showed an upregulation in the expression of NF-кB activity (1.97 ± 0.23 and 2.48 ± 0.09-fold change, respectively) compared to the Normal group basal levels (1.10 ± 0.11-fold change). Meanwhile, suppression in the activity was observed in the STD and TSE groups 0.43 ± 0.09, and 0.09 ± 0.04-fold change respectively) which was significant to the Normal group. Furthermore, a significant decrease in the expression levels of NF-кB in the STD, TSE, and MHF (0.64 ± 0.29-fold change) was seen when compared to STZ after treatment.

Nrf2 expression levels in the HFD, STZ, and TSE groups were significantly downregulated (0.38 ± 0.06, 0.23 ± 0.03, 0.33 ± 0.02-fold change, respectively) compared to the Normal group basal level (0.69 ± 0.02-fold change). Significant upregulation was observed in the STD and MHF groups (0.58 ± 0.06, 0.57 ± 0.01-fold change) when compared with the STZ group.

NQO1 expression levels were significantly decreased in the HFD, STZ, and TSE groups (0.46 ± 0.03, 0.18 ± 0.03, and 0.34 ± 0.12-fold change, respectively) compared to Normal basal level (1.14 ± 0.14-fold change), while an increased expression level was observed in STD (0.18 ± 0.03-fold change) and MHF (0.77 ± 0.05-fold change) compared to the STZ group.

Protein levels of BDNF expression were significantly decreased in the HFD and STZ groups (0.15 ± 0.00, and 0.04 ± 0.01-fold change respectively) compared to Normal basal levels (0.63 ± 0.01basal levels); however, treatment with STD, TSE, and MHF evidently increased the expression levels (0.24 ± 0.02, 0.20 ± 0.01, and 0.47 ± 0.02-fold change respectively) significant to the STZ group.

## Discussion

Accumulating evidence suggests that T2DM and Alzheimer’s disease share common pathological mechanisms. Furthermore, cognitive impairment and dementia are increasingly recognized as important complications of diabetes mellitus (Akter et al. 2011; Pasquier et al. 2006; Roriz-Filho et al. 2009). We compared TSE with MHF (a phytopharmaceutical formulation positioned in the market for effective management of T2DM) for their potential in reducing blood sugar levels and alleviating diabetes-associated cognitive defects. Pioglitazone was used as standard as it displayed a time- and dose-dependent protective effect against dementia in patients with diabetes (Chou et al. 2017).

The results revealed a much higher total phenolic content in MHF than TSE. Moreover, the free radical scavenging activity estimated in the DPPH assay revealed a lower IC_50_ value of MHF indicating a higher antioxidant activity than TSE (Kedare and Singh 2011). Similarly, a higher percentage of inhibition by MHF in FRAP assay demonstrated a higher reducing ability of MHF than TSE. This enhanced antioxidant property of MHF may contribute to its potential therapeutic effects in controlling cognitive impairment as it is also reported that cognitive impairment in diabetic rats was associated with increased reactive oxygen species (ROS) levels and reduced antioxidant levels (Zilliox et al. 2016). Increased ROS generation activates multiple cellular pathways associated with neural injury and cerebral damage (Giacco and Brownlee 2010).

Animals fed with the high-fat diet for 8 weeks in the HFD group exhibited a substantial increase in body weights compared with the normal group. However, this increase in body weight may not necessarily indicate obesity, but rather reflect metabolic alterations associated with high-fat diet feeding. The marked reduction in body weight observed after treatment with MHF and TSE may be attributed to the hypolipidemic activity of these agents (Nabeel et al. 2020).

On the 50th day, that is, after 21 days of STZ for proper induction and stabilization of the glucose levels, the animals in STZ, STD, TSE, and MHF groups showed a significant increase in blood glucose levels compared to the Normal and HFD group signifying induction of hyperglycemia with STZ. Further, the diabetes induction in the HFD-STZ group resulted in a significant increase in ELT, the number of errors, and more complicated search strategies in the track map in the Barnes maze; and a decreased PI in NORT compared to the Normal group, which indicated impairment in cognitive function in diabetic animals. This is in line with previous reports (King et al. 2013; Cordner and Tamashiro 2015; Vandal et al. 2014; Xourgia et al. 2019). The number of Errors committed reflected the ability of the rats to reach the escape hatch without multiple exploration or nose dips into the other dummy holes on the Barnes maze platform. A complicated search strategy translated to a higher ELT and the number of errors committed by the animal on the Barnes maze.

Behavioral assessment in the present study was performed using the Barnes maze and novel object recognition test, both of which are widely used paradigms for evaluating spatial learning and recognition memory in rodent models (Gee et al. 2023; Lueptow 2017). The Barnes maze offers advantages over water-based tests such as the Morris water maze, as it minimizes stress associated with forced swimming and relies on the natural exploratory behavior of rodents (Bertolli et al. 2024). Therefore, it is considered suitable for assessing spatial learning in metabolic disease models where stress-related confounding effects may influence performance.

Furthermore, during behavioral testing, animals did not exhibit visible locomotor impairments or abnormal exploratory behavior that could interfere with maze performance. Thus, the observed differences in escape latency time and error frequency are likely attributable to cognitive impairment associated with diabetes rather than alterations in locomotor activity or anxiety-related behavior.

The treatment of diabetic animals with STD-pioglitazone, MHF, or TSE for 4 weeks significantly reduced glucose levels compared to Normal suggesting their antidiabetic potential (Mitra et al. 1995; Liu et al. 2013). Similarly, a significant reversal in cognitive decline was observed in animals after 12 weeks of treatment with MHF as compared to the STZ group, as evidenced by the decreased ELT and number of errors, a less complicated search strategy in the track map, and significantly increased PI. This may be attributed to its nootropic activity mediating through PPARγ agonist activity and anti-inflammatory activity (Xourgia et al. 2019; Rojanathammanee et al. 2013). Here, we report the effect of MHF (Diabecon) in diabetes-associated cognitive decline (DACD) and its potential reversal. On the other hand, TSE failed to reverse the cognitive impairment as evidenced by increased preference towards the familiar object with a PI of 48.54 ± 4.66% in the HFD-STZ model. Similar results were reported following the treatment using Ethanol Garlic Extract in the T2DM model of rats (Semuyaba et al. 2017).

The estimation of antioxidant enzymes revealed that, compared to the normal group, untreated diabetic animals in the STZ group showed lower levels of superoxide dismutase and catalase, which are key enzymes involved in the primary defense against oxidative stress. These findings agree with the previous report (Liu et al. 2013). The treatment with MHF significantly reversed the diabetes-induced alterations in endogenous antioxidant enzymes, indicating its promising antioxidant potential. To the best of our knowledge, this is the first study reporting the effect of MHF-Diabecon in diabetes-induced oxidative stress. MHF may contribute to reducing free radical generation during diabetes by reducing the production of hydrogen peroxide and oxygen. This would maintain the homeostasis of the endogenous antioxidant enzymes, thereby elucidating its protective action in the diabetic brain (Uttara et al. 2009; Kurutas 2016). The decreased level of oxidative stress may finally result in a reduced level of inflammation in the brain. These findings agree with a previous report in T2DM rats treated with *Scopariadulcis* (Pari and Latha 2004). Like the above cognitive parameters and the blood glucose levels, the TSE treatment failed to recover diabetes-induced oxidative stress.

Estimation of Glutamate and GABA levels, the neurotransmitters associated with cognition, in the hippocampus of the obese diabetic model showed a significantly lower decrease in glutamate and higher GABA compared in STZ-induced diabetic animals than the Normal group, which is aligned with the previous report (Xourgia et al. 2019; Kumar Datusalia and Sunder Sharma 2016). MHF treatment significantly restored glutamate and GABA levels, suggesting improved neurotransmitter balance associated with cognitive function. According to our knowledge, this is the first study to report the effect of MHF- Diabecon in diabetes-induced alterations in glutamate-glutamine (Glu-Gln) homeostasis. MHF might have aided in maintaining the Glu-Gln cycle homeostasis by upholding the Glutamine synthase and Glutaminase enzymes (Cruzat et al. 2018). TSE treatment was only effective in elevating the Glutamate levels, although there was a decrease in the GABA levels but not significant compared to the STZ control group.

Further, in ELISA estimation the STZ control group showed a significantly decreased level of hippocampal insulin compared to the Normal group, which increased significantly upon treatment with MHF. The observed increase in hippocampal insulin levels following MHF treatment may contribute to improved neuronal metabolism and synaptic plasticity. Insulin signaling within the hippocampus has been reported to influence cognitive processes by modulating neuronal survival, synaptic function, and neurotrophic factors such as BDNF. Therefore, the restoration of hippocampal insulin levels observed in the present study may be associated with the improvements in cognitive performance (Hu et al. 2013). However, TSE treatment did not show any significant increase in the brain insulin level.

The hippocampal homogenates of the diabetic animals showed significantly elevated levels of TNF-α, IL-6, and IL-1β when compared to the Normal group, which agreed with previous reports (Wang et al. 2019a, b; Akter et al. 2011). MHF treatment significantly reduced these pro-inflammatory markers, viz., TNF-α, IL-6, and IL-1β, compared to the STZ control group, revealing its potential anti-inflammatory properties and efficacy in reversing DACD. According to our knowledge, this is the first study to report the reversal of inflammatory mediators in DACD. TSE also demonstrated significantly reduced TNF-α, IL-6, and IL-1β levels compared to the STZ control, which might be attributed to its anti-inflammatory, antioxidant activity (Kinattingal et al. 2016).

The molecular markers involved in the antioxidant and anti-inflammatory homeostasis system, such as Nrf2, NQO1, NF-κB, and BDNF were estimated by Western blot in the hippocampal homogenates (Hwang et al. 2011). In the current study, the diabetic animals expressed a significant upregulation of NF-κB in the hippocampal homogenates of the STZ control groups compared to the Normal group and significant downregulation of Nrf2 and NQO1 marker enzymes. This result is in line with previously reported data which stated an increase in the expression of NF-κB in hippocampal tissues of T2DM mice (Wang et al. 2019a, b). This diabetes-associated imbalance in the antioxidant, and anti-inflammatory homeostasis might be linked to the cognitive decline observed in these animals (Pu et al. 2018).

MHF treatment at a dose of 500 mg/kg bwt was associated with a reduction in NF-κB expression and an increase in Nrf2 and NQO1 expression compared to the STZ control group. The result agrees that Nrf2 -mediated antioxidant response is considered important for pancreatic β -cell protection against ROS/RNS and xenobiotics (Tu et al. 2019; Yang et al. 2012). Furthermore, Nrf2 is known to play a crucial role in neuroprotection by activating the antioxidant defense system, lowering inflammation, and balancing protein homeostasis (Singh et al. 2021). Furthermore, STD (Pioglitazone) treatment at a dose of 20 mg/kg bwt significantly downregulated NF-κB expression and upregulated Nrf2 and NQO1 expression compared to the STZ control group. This is in concurrence with previous findings where pioglitazone, a PPARγ agonist exhibited a neuroprotective effect via Nrf2/ARE-dependent HO-1 pathway (Zakaria et al. 2019).

However, the present study primarily demonstrates associations between biochemical and behavioral changes, and further mechanistic investigations would be required to establish direct causal relationships between these signaling pathways and cognitive improvement.

Although the HFD–STZ-induced diabetic rat model is widely used to reproduce metabolic disturbances associated with type 2 diabetes, it may not fully replicate the complex pathophysiology of diabetes-associated cognitive decline observed in humans. Human diabetic cognitive impairment develops over prolonged periods and involves multiple factors including chronic metabolic dysregulation, vascular complications, and lifestyle-related influences that cannot be completely reproduced in experimental animal models (Marino et al. 2023; Ab-Hamid et al. 2023). Therefore, while the present findings provide valuable mechanistic insights into the neuroprotective potential of the investigated interventions, caution should be exercised when extrapolating these results directly to clinical conditions.

Despite these limitations, these findings highlight the therapeutic potential of antioxidant-rich herbal formulations in mitigating diabetes-associated cognitive impairment. The observed modulation of oxidative stress, inflammatory signaling pathways, and neurotrophic markers such as BDNF suggests that multi-targeted phytotherapeutic approaches may provide complementary strategies for managing neurological complications associated with diabetes. However, further studies including detailed mechanistic investigations and well-designed clinical trials will be required to confirm the translational relevance of these findings in human populations.

## Conclusion

Our data suggests that progressive hippocampal neurodegeneration occurs in HFD-STZ-induced T2DM, as confirmed by a significant decrease in BDNF and insulin levels leading to cognitive decline. MHF having antioxidant, anti-inflammatory, and antidiabetic activity was able to reduce oxidative stress and inflammation and, in turn, combat cognitive decline by increasing the hippocampal levels of BDNF and insulin, whereas TSE was unable to alleviate cognitive decline. Elevated endogenous antioxidant enzyme levels, suppression of lipid peroxidation, and inflammatory mediators by downregulating the NF-кB pathway were useful in ameliorating cognitive decline in T2DM animals.

HFD: high-fat diet; MHF: Marketed Herbal Formulation; NORT: novel object recognition test; STD: standard—Pioglitazone; STZ: streptozocin; TSE: *Tamarindus indica* Linn. Seed Extract.

## Abbreviations

T2DM: Type 2 diabetes mellitus
DACD: Diabetes-associated cognitive decline
AD: Alzheimer’s disease
MCI: Mild cognitive impairment
FDA: Food and drug administration
OHA: Oral hypoglycemic agents
MHF: Marketed herbal formulation (Diabecon^®^)
TSE: *Tamarindus indica* seed extract
STZ: Streptozotocin
OGTT: Oral glucose tolerance test
SD: Sprague Dawley (rats)
IP: Intraperitoneal
DW: Distilled water
i.g.: Intragastric / oral gavage
OG: Oral glucose
MST: Morris water maze test
NOR: Novel object recognition test
MWM: Morris water maze
SRM: Spatial reference memory
ORM: Object recognition memory
SOD: Superoxide dismutase
GSH: Reduced glutathione
MDA: Malondialdehyde
AChE: Acetylcholinesterase
ROS: Reactive oxygen species
H&E: Hematoxylin and eosin
SPSS: Statistical package for social sciences
ANOVA: Analysis of variance
SEM: Standard error of mean
BGL: Blood glucose level
LTP: Long-term potentiation
